# "The Hospital" Medical Book Supplement.—No. XVII

**Published:** 1909-04-24

**Authors:** 


### "THE HOSPITAL" MEDICAL BOOK SUPPLEMENT.—No. XVII.

CONTENTS.

**Medicine—**
An Index of Treatment ... 109
Diseases of the Digestive Canal ... 109
Soured Milk and Pure Cultures of Lactic Acid Bacilli in Treatment ... 109
**Surgery—**
Estimation of the Renal Function in Urinary Surgery ... 110
Manual of Operative Surgery ... 110
Transactions of the Tenth Annual Meeting of the American Proctological Society, 1908 ... 110
**Diseases of Nose, Throat and Ear—**
Manual of Diseases of the Ear, including those of the Nose and Throat in Relation to the Ear ... 111
Text-Book of Diseases of the Nose, Throat, and Ear, for the Use of Students and General Practitioners... 111
**Miscellaneous Items—**
The Medical Annual, 1909 ... 112
The Causation of Sex ... 112
Life Insurance and General Practice... 112
The Edinburgh Stereoscopic Atlas of Obstetrics... 112

### MEDICINE.

An Index of Treatment. By Various Writers. Edited by Robert Hutchinson and H. Stansfield Collier. Fourth Edition. (Bristol: John Wright and Sons. Pp. 926. Price, 21s. net.)

The fact that this book has reached a fourth edition in the space of sixteen months speaks for the public estimation in which it is held. And indeed its popularity is well merited, for it fills, and fills efficiently, a relatively empty pigeon-hole in the vast library of medicine. Advancing knowledge in matters of diagnosis and pathology has left the poor student with scanty time to devote to the learning of treatment, except in bald outline; and this although everything else that he learns is in its essence but ancillary to treatment. That is to say, the end, under existing circumstances of student-life, is made subordinate to the means; a topsy-turvy state of affairs, but one not easily avoided. This applies more particularly to medical than surgical matters, for it is undeniable that surgical treatment is better taught than medical, chiefly because it is so much more defined and circumscribed. The minor medical ailments, on the other hand, which bulk so large in practice, inevitably meet in hospitals with a somewhat cavalier reception, partly because they are overshadowed by graver ones, partly because the mass of work leaves no time for their detailed consideration. In consequence a man who knows well enough, it may be, the technique of the operation for intestinal anastomosis (which he is quite unlikely to be called upon to perform) may well go into practice ignorant of the way to manage a neurasthenic patient who presents some intrinsically trifling symptom, or a baby that is constipated and keeps the household awake all night. And these are certain to meet him. The minuter details of treatment must in the nature of things be learnt by experience, but such a book as this, which offers within a reasonable compass a *précis* of the most recent treatments compiled by masters in their own subjects, cannot fail to be an asset to every practitioner. Now and then, as is to be expected in a book from many pens, one comes across contradictory opinions. For example, in Dr. Milne Bramwell's temperate and judicial article on "Hypnotism" we read: "As far as my experience goes, the employment of hypnotism by medical men acquainted with the subject is absolutely devoid of danger." In the succeeding article upon "Hysteria," an excellent one, from the pen of Dr. Risien Russell, we are told that hypnotism is a most potent agent in the treatment of the affection, but that "it labours under the disadvantage that it may be responsible for increasing the mental instability, and even for producing more serious mental derangement." But such antagonisms do not detract from the value of the articles, for their existence merely evidences the fact that upon the disputed points expert opinion is still labile. Decidedly the book is a most valuable one.

Diseases of the Digestive Canal. By Dr. Paul Cohnheim. Edited and translated from the second German edition by Dudley Fulton, M.D. (J. B. Lippincott Co. Price, 16s. net.)

This volume is very clearly printed in large type, and it is written in a dogmatic style which will commend itself to many practitioners. It restricts itself to diseases of the œsophagus, the stomach, and the intestines, without entering upon those of organs such as the liver and pancreas, whose ducts open into the alimentary canal. It deals at length with laboratory methods for investigating gastric and intestinal products, and it also gives clear accounts of symptoms, differential diagnosis, and medicinal and other non-operative treatment. As is almost inevitable in any such work, there are several points in Cohnheim's book with which we and many other readers do not agree. At the same time, it contains many helpful suggestions of which practitioners will be glad. It is true that it deals very shortly with some subjects which it might be expected would be described at length. Appendicitis, for example, is dismissed in about five pages. So rapidly is every branch of medicine and surgery advancing nowadays, that any translation is almost certain to be already a little out of date by the time it is in print. This applies, we think, to the present volume. Nevertheless, upon the whole it is a work which those particularly interested in diseases of the digestive canal will be glad to refer to, and possibly to own.

Soured Milk and Pure Cultures of Lactic Acid Bacilli in Treatment. By George Herschell, M.D. 2nd impression. (London: H. J. Glaisher. Chicago: Keener and Co. Pp. 32. Price, 1s. 6d. net.)

This is to all intents and purposes an essay or paper in book-form upon the cure of diseases by lactic acid ferments, written in the enthusiastic and exaggerated style so familiar in connection with new varieties of treatment. The booklet is divided into three parts. The first treats of the main points about auto-intoxication and intestinal putrefaction. The second part deals with the selection and preparation of lactic acid ferments for use in practice. The author finds faults in the use of commercial Yoghourt prepared with the Maya ferment; all commercial soured milks; liquid cultures of bacilli for internal use; liquid cultures in tubes for souring milk; and many of the various dried cultures on the market. He insists upon the importance of the Bulgarian bacillus of Boucard, with or without a strepto-bacillus, which may be added to render the taste more agreeable by preventing the saponification of the fat of milk. He lays down practical rules for the selection of the tablet or powder that is best. He gives his tablets by the mouth, not hypodermically, which is a favourable point. The third part considers the various maladies in which, according to the author, the Bulgarian bacillus treatment is indicated.

### SURGERY.

Estimation of the Renal Function in Urinary Surgery. (By J. W. Thomson Walker. (London: Cassell and Co. 1908. Pp. 275. Price 6s.)

There is a great deal in Mr. Thomson Walker's book that must be read with interest by all who practise urinary surgery. For one thing it affords a short cut to acquaintance with the literature of the subject; for another it offers practical instructions for the application of many unfamiliar diagnostic or discriminative procedures. Let us add, too, that in the description of cystoscopic manipulations and the use of segregators, all that could be expected within the limits of so few pages is well accomplished. At the same time the book does not entirely carry conviction. Many of the methods for estimating renal function, clever and ingenious as they are, introduce so many variables that the problem admits of only the most dubious solution. To take as an example the value of evidence as to renal function derived from observations of the time of appearance of a colouring substance in the urine after exhibition of a drug. There is first of all the uncertainty due to wide limits in the rate of elimination by "healthy" kidneys; then there is the opportunity for error arising out of deputed observation in the collection of the urine; again, there are the difficulties of determining the collateral effects of diet, temperature, and the psychic condition of the patient. As matters stand at present it is a question whether the game is worth the candle. Of course the author does not claim anything like perfection for the methods he describes and advocates; he has, indeed, a chapter on fallacies, and he disarms criticism to some extent, in his preface, by intimating that the present work is preliminary to further study. We must not be understood to detract from the value of this initial work at an important subject, recorded in this book if we say that his further experience will lead him to discard a good many methods now on their trial with him, as cumbersome and insufficiently productive. About half the book is taken up with methods for the differentiation of the shares of the two kidneys in the total urinary function of any given case, and is so obviously founded on continuous and extensive practice that it will make a useful manual for those who desire to become expert in the use of instruments that, in any but expert hands, are misleading, if not dangerous. The volume is well printed, well illustrated, has a satisfactory index, and, it may be mentioned, gives ample acknowledgment of the source of information from other workers in footnotes.

Manual of Operative Surgery. By H. J. Waring, M.S., M.B., B.Sc., F.R.C.S., Senior Assistant Surgeon St. Bartholomew's Hospital. Third edition. Pp. 750 + xxxii. with 521 figures, several in colours. (London: Henry Frowde and Hodder and Stoughton. Oxford Medical Publications. Price 12s. 6d. net.)

Mr. Waring's excellent "Manual of Operative Surgery" has for the past ten years occupied a special place among the many works devoted to this subject, and a third edition scarcely needs a detailed description to introduce it to the medical profession. Although small in size, its range of information is quite sufficient for the needs of most students and general practitioners; while for those engaged more especially in operative work it provides a useful summary of all ordinary procedures, and may be used with advantage as a compendious adjunct to the larger and more inclusive systems of operative surgery, most of which are published in several volumes at a proportionate cost. The first two editions of this manual, issued eleven and five years ago respectively, showed that it was possible to provide in one handy volume a clear, readable and practical text-book, profusely illustrated, which would be sufficient, not only for a full course of formal instruction in operations upon the dead body, but also as a useful guide to the corresponding operations as performed in the theatre on the living body. The opening chapters, devoted to preliminary generalisation, and to the preparations and equipment necessary for operations, are very much as before, and supply sound information in small compass. The new illustrations are clear and instructive, and add to the value and appearance of the volume. In spite of many necessary additions the size and weight are much as before, and the work is still entitled to be styled a "hand-book"; but it is probable that any further increase in scope and illustration, or any further elaboration of detail, however useful, would detract from the value of the work, which is essentially a manual and not an inclusive system. This is of course the main problem before the writer of small text-books. Medicine and surgery are yearly extending their range; and selection thus becomes a matter for much care and discrimination, and a large degree of common sense. The price of the present edition has been raised from 10s. 6d. to 12s. 6d. net, and although this is for some reasons to be regretted, it cannot be denied that the many additions are worth the extra two shillings, and that the third edition of Waring's "Operative Surgery" is excellent value for the money. The ophthalmic and gynæcological chapters remain for the most part the revised work of Mr. W. H. Jessop and Dr. Hubert Roberts, respectively, and will be found useful summaries of the common operative procedures in these special regions. Mr. Douglas Harmer and Mr. C. E. West have revised the chapter upon the throat and nose and upon the ear; while throughout the volume there are signs of careful revision, and the elimination of unnecessary and obsolete particulars. The new operations which have been introduced are well selected, and in various places we note that former descriptions have been made clearer and in some cases rewritten.

Transactions of the Tenth Annual Meeting of the American Proctological Society, 1908.

These transactions, at the conclusion of ten years' work, of a Society limited to the study of the anus, rectum, and pelvic colon do not readily lend themselves to connected criticism. To our mind the most valuable contribution to surgical science is the paper containing some new work on the innervation of the lower bowel. If the author's contention is verified that there are dilator as well as constrictor sympathetic nerve fibres to the internal sphin ter, the fact may well have important bearings on the physiology of the rectal function. We do not find ourselves in accord with the views on the pathogenesis of simple stricture of the bowel, as expressed by several of the contributors, who seem to lay undue stress on syphilis as the causative factor; neither do we altogether agree with the treatment advocated for these conditions. The surgical measures recommended in some cases of stricture border on the heroic, and we have nothing but admiration to express for the surgeon who removes twenty-two inches of large bowel for this condition. Malignant disease is discussed by several of the proctologists, stress being laid on the necessity of early operation, and a complete series of all cases of melanotic sarcoma in the region is inserted. A few interesting rarities are recorded as short communications, and the last paper deals with anæsthetics, but this seems to be totally out of place in such a volume.

### DISEASES OF NOSE, THROAT AND EAR.

Manual of Diseases of the Ear, including those of the Nose and Throat in Relation to the Ear. By Thomas Barr, M.D., and J. Stoddart Barr, M.B., Ch.B. Fourth Edition. (Glasgow: James Maclehose and Sons. Pp. 477 + xxvii., with 3 coloured plates and 215 illustrations. Large 8vo. Price 14s. net.)

Among the numerous manuals on diseases of the ear which have recently appeared we welcome the fourth edition of Dr. Barr's well-known work. It is a large volume and the attempt which has been made to reduce its apparent size by printing considerable portions in small type, does not tend to make its perusal easier. The size is largely due to the arrangement of the work, which has resulted in considerable repetition. In the first four chapters the characters of ear diseases are treated as a whole under the headings "Methods of Examination," "Symptomatology," "Causes of Ear Disease," and "Methods of Treatment." Thus, the micro-organisms found in aural discharges are described in the chapter on symptoms and again in that on causation; mutism has a paragraph under "Symptomatology" and a chapter to itself at the end of the volume. So, also, the subject of syringing, with illustrations of the instruments employed, is discussed in the general chapter on treatment, and this is repeated—with the pictures—when describing the special treatment of suppuration.

With regard to the 35 pages on diseases of the nose and throat in relation to the ear, we cannot but feel that a work of this nature should either be a complete manual of diseases of the ear, nose and throat or else omit these latter altogether. Such a description cannot be thorough and complete, and the practitioner who desires to be fully equipped to deal with disease of these regions must of necessity have recourse to special works on the subject. The same remarks apply with greater force to the six pages devoted to general anæsthesia in operations on the ear, nose and throat. We must refer to one other point and deprecate the mention in a text-book of special and proprietary preparations of well-known drugs, especially coupled with the names of their manufacturers. In the description of local anæsthesia by cocaine and adrenalin it would appear unnecessary to mention a particular combination of these drugs under a coined title with the maker's name, and when discussing the injection of pilocarpine nitrate there is no need to name the preparation of a well-known firm. As a final grumble, the space seems to us to be rather unevenly allotted; in a work of this size three pages are a scanty allowance for the entire description of so common and important an affection as otosclerosis, and we could wish that more space had been devoted to diseases of the internal ear, which are dismissed in twenty pages, and that the reactions to the tuning-fork tests in the various forms of deafness had been dealt with somewhat more fully. We have commented at length on what appear to us to be defects in the book and which are, after all, chiefly matters of arrangement, but we do not wish to appear unappreciative of its many excellent and sterling good qualities. The opinions expressed are thoroughly sound, the work has been written with great care and shows evidence everywhere of much personal experience.

The after-treatment of operations is particularly well described in this volume, and this chapter should prove most useful to every surgeon and practitioner who has to deal with these cases; we have also nothing but praise for the very full and thorough manner in which the intracranial complications are discussed. The numerous illustrations are for the most part excellent, though several are repeated in different parts of the book, and the 45 new pictures of various conditions of the drum have been beautifully reproduced by the four-colour process and are worthy of the highest praise. The index is unusually good, and there is also a bibliography.

Text-book of Diseases of the Nose, Throat, and Ear, for the Use of Students and General Practitioners. By Francis R. Packard, M.D. (Philadelphia and London: J. B. Lippincott Company. Pp. 369 + xiv., with 3 plates and 135 illustrations. Price 15s. net.)

This is a most disappointing book; we took it up with a feeling of pleasant anticipation, for it is well printed on good paper and the illustrations are for the most part excellently reproduced. But the work is very far from thorough; much space is occupied by irrelevant matter, and information concerning points of detail and difficulty on which the practitioner naturally looks for guidance in a work of this kind is conspicuous by its absence. To take a few instances: in the chapter of 15 pages devoted to diseases of the accessory sinuses of the nose, it is stated that the earliest subjective symptom of antral disease is pain—no attempt is made to separate acute and chronic inflammations of this cavity, and no mention whatever is made of latent sinus disease. The author describes a "radical operation" which consists in opening through the canine fossa and packing with gauze until the wound has healed by granulation; removal of the antro-nasal wall is not even discussed, and no allusion is given to advances in the treatment of this cavity which have been made in the last ten or fifteen years. Laryngeal tuberculosis receives just over three pages, with no reference whatever to the work done by Heryng, Krause and others on the local treatment of this affection, nor mention of any treatment for the dysphagia except the use of cocaine. Under "Tumours of the Larynx" there is no mention of the difficulties met with in dealing with multiple papillomata in children, and the only recommendation for treatment is to perform tracheotomy and leave the case alone. The most characteristic symptom of paralysis of the recurrent laryngeal nerve is said to be aphonia, but then the cord is said to lie midway between adduction and abduction, and the author is apparently unaware that abductor paralysis is the first result of pressure on the recurrent nerve.

Bilateral paralysis is said to be usually due to "a lesion in the central nervous system," but it is not stated in which nervous diseases the affection occurs; indeed, there is nowhere a complete or connected account of the causes or significance of the diseases described. Diseases of the ear are treated in the same casual manner; cerebral abscess is dismissed in nine lines, while diseases of the auricle receive nine pages; there is no illustration of the normal or retracted drum, but seven large pictures of malformations of the auricle, three of which are cases of adherent lobule. Indeed, the illustrations show a peculiar irrelevancy, as may be exemplified by the fact that of the three coloured plates two are photographs of an anatomical preparation of the cranial nerves, and the third shows a child with a vaccination sore on the nose and is accompanied by a picture of the vesicle on the arm from which the infection was derived! It will serve no useful purpose to prolong the unpleasant task of criticism when every chapter shows similar characteristics.

### MISCELLANEOUS ITEMS.

The Medical Annual, 1909. (Bristol: J. Wright and Co. Price 8s. 6d. net.)

The twenty-seventh yearly issue of this well-known publication maintains the catholic and comprehensive character of its predecessors. The magnitude of this highly condensed account of the medical researches of the past year is a tribute to the industry, if to nothing more, of the medical profession, and also to the vigilance of the editorial staff. Difficult as it must be to keep within limits an annual whose natural tendency is to expansion, the present volume is no bulkier than its recent forerunners; this, however, has been achieved by using thinner paper, so that the actual reading matter is greater. Criticism in detail of the latter is impossible, but most of it is admirable; omissions are few indeed, but certain contributions might with advantage have been more liberally blue-pencilled. Various special features which have assisted the popularity of the "Medical Annual" in the past are once more noticeable. Especially to be commended is the large number of illustrations and coloured plates, which are well selected, excellently reproduced, and increase materially the value of the text. In the introductory chapter on therapeutic progress and elsewhere is still to be noted, though we are glad to think to a less extent than last year, a tendency to include unconfirmed claims made in German and American papers on behalf of various protected and proprietary products, some of which have been already discredited. The difficulty, no doubt, is to choose between undue scepticism, which means a delay of a year before a new remedy can be mentioned; and undue credulity, which involves the inclusion of much that is disproved before the "Annual" is printed. Considering how very few of these new drugs (or new names of old ones) attain any general recognition, the first alternative is much to be preferred. The editors have once more included somewhat lengthy essays on special subjects by distinguished foreigners. This year Professor Béraneck outlines his views on the use of his tuberculin, and Professor Lucas-Championnière explains his teachings on the treatment of fractures. Interesting as these contributions are of themselves, it may perhaps be questioned whether this particular part of the scheme of policy is wise, considering the growing bulk of the rest of the volume. However, the publishers have probably realised by this time that they cannot please everybody; but if their "Annual" pleases the mass of the profession as much as it pleases us, we feel sure that its circulation must be extremely gratifying to them.

The Causation of Sex. By E. Rumley Dawson. Pp. 190. 21 illustrations; index. (H. K. Lewis. 6s. net.)

Enthusiasm is the first ingredient requisite for success in any scientific investigation, and Dr. Rumley Dawson is by no means deficient in this respect. Occasionally we find his remarks somewhat too reiterative, though we fully sympathise with a man who hopes to have made a great discovery. Even the great Lamarck was not above emphasising the importance and originality of his own work. Dr. Rumley Dawson has enunciated an interesting theory to account for the sex distinction in the genus homo. He maintains (1) that the male pronucleus has nothing whatever to do with sex causation; (2) that male children come from ova which dehisce from the right ovary, female from the ova of the left; (3) he is inclined to belief in polyspermy in man, as opposed to the ordinary view that one spermatozoon alone penetrates into the ovum. Generally, the facts well arranged and the theory is certainly presented as favourably as could be desired by its author. But when he discusses the more controversial side of the question, the objections which he cites appear rather like wisps from a man of straw. The argument, for example, that because more boys are born than girls, and because the right ovary is larger than the left, therefore right ovaries produce boys, is quite unsound logically. Nor do we find anywhere the suggestion that the fact that the right ovary is larger is simply in accordance with the more general right-handedness of men and women. The book on the whole is suggestive and ingenious.

Life Insurance and General Practice. By E. M. Brockbank, M.D., F.R.C.P. Pp. xiv. + 288. (London: Henry Frowde, Hodder and Stoughton. 1908. Price 7s. 6d. net.)

Dr. Brockbank's manual fulfils admirably the purpose for which it has been written. There are few, if any, problems arising in the course of examinations for life insurance which he does not consider, and his discussion both of facts and their meanings is invariably concise, lucid, and practical. Hence, as a guide to those who undertake the responsible work of medical examiner, this book may be very heartily commended. The earlier sections are devoted mainly to the methods of examination. In them are to be found numerous suggestions and hints which show the experienced hand and brain, and which are of great practical value. In the later part of the work detailed attention is paid to the question of impaired but yet insurable lives, and the various problems which have to be met are duly considered. It is these problems which constitute the special difficulties of life-insurance work, and to meet these difficulties Dr. Brockbank's pages afford reliable and substantial help.

The Edinburgh Stereoscopic Atlas of Obstetrics. Edited by G. F. Barbour Simpson, M.D., and Edward Burnet, M.A., M.B., with a Preface by Sir Halliday Croom, M.D. Section III. (London: The Caxton Publishing Co. In four sections, 84s. net complete.)

The third section of this atlas of obstetrics maintains the high degree of excellence attained by the first two sections. Dealing with the anatomy of presentations and mechanisms of labour in the second and third stages, the photographs present in a lifelike manner the various phenomena of labour, which can be shown by pictures. The method of use of the axis traction forceps, the Walcher position, and some pictures of post-partum uteri complete the twenty-five subjects dealt with.

